# Direct measurement of vagal tone in rats does not show correlation to HRV

**DOI:** 10.1038/s41598-020-79808-8

**Published:** 2021-01-13

**Authors:** Joseph T. Marmerstein, Grant A. McCallum, Dominique M. Durand

**Affiliations:** Department of Biomedical Engineering, Case Western Reserve University, Cleveland, OH USA

**Keywords:** Autonomic nervous system, Biomedical engineering

## Abstract

The vagus nerve is the largest autonomic nerve, innervating nearly every organ in the body. “Vagal tone” is a clinical measure believed to indicate overall levels of vagal activity, but is measured indirectly through the heart rate variability (HRV). Abnormal HRV has been associated with many severe conditions such as diabetes, heart failure, and hypertension. However, vagal tone has never been directly measured, leading to disagreements in its interpretation and influencing the effectiveness of vagal therapies. Using custom carbon nanotube yarn electrodes, we were able to chronically record neural activity from the left cervical vagus in both anesthetized and non-anesthetized rats. Here we show that tonic vagal activity does not correlate with common HRV metrics with or without anesthesia. Although we found that average vagal activity is increased during inspiration compared to expiration, this respiratory-linked signal was not correlated with HRV either. These results represent a clear advance in neural recording technology but also point to the need for a re-interpretation of the link between HRV and “vagal tone”.

## Introduction

The autonomic nervous system governs subconscious control and sensing of visceral organ activity. It has recently become a focus for novel therapeutic technologies, such as “bioelectronic medicine”^1^. The vagus nerve is the largest autonomic nerve, innervating nearly every organ in the body^2^. Its widespread innervation has made the vagus nerve a target for therapeutic interventions, with vagus nerve stimulation therapies being used to treat many diseases, from epilepsy to rheumatoid arthritis to depression^3–7^.

While vagus nerve activity has not been directly measured in these diseases, heart rate variability (HRV) is a clinical measure presumed to assess vagal activity, often termed “vagal tone”^8^. Abnormal vagal tone has been associated with many severe conditions such as diabetes, heart failure, and hypertension^9–15^, and with worse patient outcomes^16–18^. Yet, vagal tone has only been measured indirectly until now^19^. Additionally, the physiological mechanism underlying HRV has been debated in literature: some believe HRV to be representative of overall vagal activity^18–21^, while others claim HRV is only driven by cardiac vagal activity, or a combination of cardiac vagal efferents and baroreceptor afferents^22–24^. HRV, whether representative of overall vagal activity or only a small subset of vagal fibers, may also be driven by phasic vagal activity related to respiration, rather than the tonic level^22,25,26^. Additionally, there are a large number of metrics that can be used to measure HRV, including time-based, frequency-based, and non-linear measures^19,27^. Unfortunately, there is not a clear consensus on the best HRV metric, leading to a lack of clarity when it comes to interpreting results. This is further complicated by the fact that many of these metrics are highly correlated with each other^19^. Here, direct, simultaneous measurement of vagal activity and heart rate are used to determine if any of these HRV metrics are actually related to tonic or phasic vagal activity.

Despite the importance of the vagus nerve, very little is understood about the role it plays in normal and pathological function, and how vagal stimulation can improve outcomes in some patients^28^. Previous physiological studies of the vagus nerve have been limited, due to difficulties recording chronic signals in the vagus (and other small autonomic nerves)^29–32^. Extraneural cuff electrodes, while useful for stimulation, suffer from small signal-to-noise ratio, and can be difficult to implement in small autonomic nerves such as the rat vagus nerve (diameter ~ 250 to 500 µm). Intraneural electrodes can provide higher signal-to-noise ratios, but many suffer from poor chronic stability and low durability due to the large size and low flexibility of the electrodes relative to the neural tissue. As a result, recordings from the vagus nerve have been largely limited to non-survival experiments, including both whole nerve recordings (using hook or cuff electrodes), or recordings from single fibers. We previously reported on the development of a novel recording technique for chronic intrafascicular recording using carbon nanotube yarn (CNTY) electrodes, resulting in high-quality signals in the rat vagus nerve for up to 10 weeks after implantation^33^. CNTY electrodes are small (10 µm diameter), and highly flexible with low-impedance, creating an axon-like interface that allows high-SNR recordings in small autonomic nerves.

In this study, we further refined the CNTY chronic recording method, improving electrode manufacture, surgical technique, vagal signal processing, and developing a technique for chronic recording in awake, behaving animals. We report here the first direct chronic recordings of natural vagal activity in rats, and results which elucidate the relationship between HRV, true vagal tone, and respiratory vagal activity.

## Results

### Chronic recording in the vagus nerve

**Figure 1:**
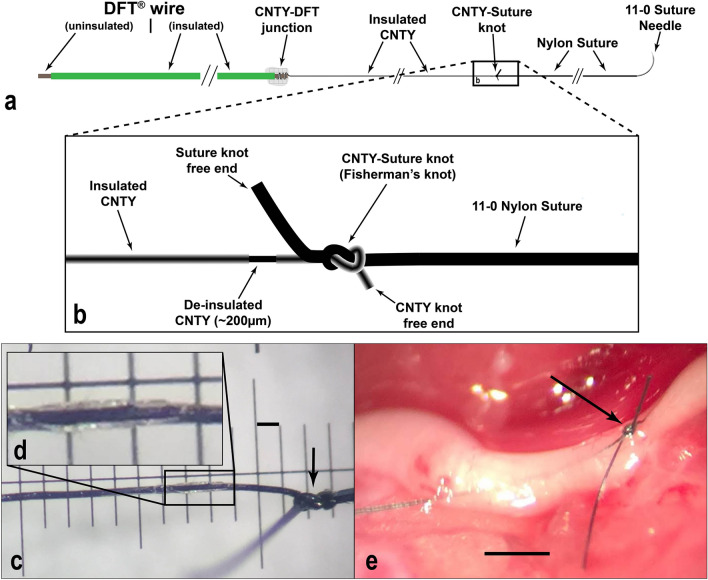
Implantation of CNTY electrodes using the suture insertion method. In all images, the CNT is on the left, while the suture is on the right. (**a**) Not-to-scale schematic showing the entire electrode assembly. (**b**) Detailed schematic of the de-insulated CNTY and the fisherman’s knot which secures the electrode to the suture. (**c**) CNTY, suture, and de-insulated portion of the electrode. Scale bar represents 100 µm. A small section of parylene-C is removed by laser. Arrow shows the fisherman’s knot which secures the CNTY to the suture, and helps to keep the electrode in place after implantation. (**d**) Inset image shows the removal of ~ 200 µm parylene-C insulation. (**e**) Implanted CNTY. Scale bar represents 500 µm. Arrow shows the fisherman’s knot. After implantation of two electrodes, the electrodes and nerve are covered with fibrin glue to secure the electrodes in place.

**Figure 2:**
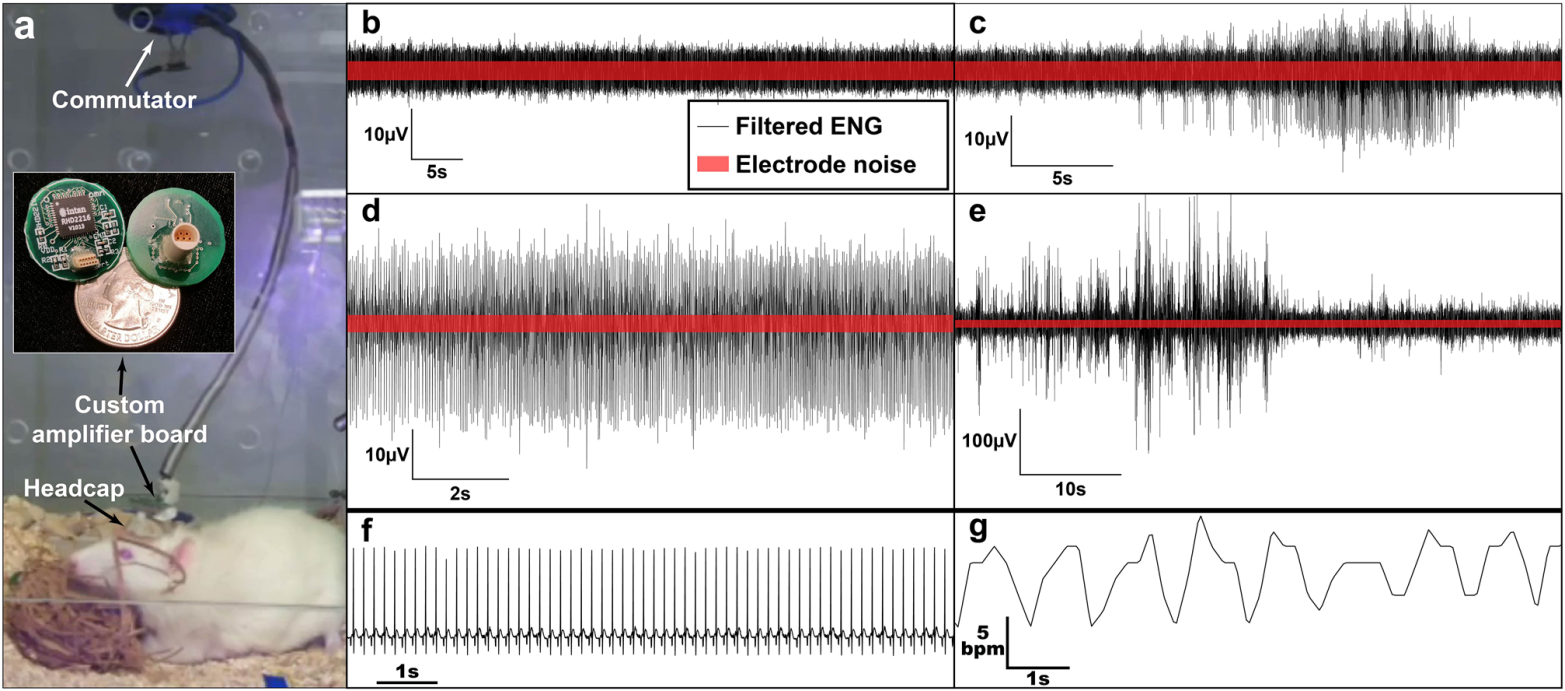
Awake recording setup and sample data. Red bars show electrode noise levels (calculated from electrode impedance and input amplifier noise). (**a**) Chronic non-anesthetized recording setup. The rat has a connector mounted to the skull headcap, where a custom amplifier board (inset image) is connected. The cable is routed to a commutator which allows the rat to move around without the cable becoming tangled or twisted. A metal spring is used to protect the cable from being chewed on. This setup can be used for 24/7 continuous recording (purple glow is glare from an infrared-equipped camera). (**b**) Quiet baseline, where there are few/no spikes significantly above the baseline. This is the most common type of activity observed, especially under anesthesia. (**c**) Intermittent spiking, where spikes are observed but appear and disappear quickly. (**d**) Spike bursting, where long bursts of spikes (of varying amplitude and firing rate) occur for periods of 30 s or longer. (**e**) Large intermittent spiking, where very large spikes (> 100 µV pk-pk) are observed in short bursts. This is most common without anesthesia but occurs with anesthesia as well. (**f**) Simultaneously recorded ECG**.** (**g**). Heart rate calculated from ECG.

Chronic recording in the rat vagus nerve has proven challenging, with the majority of vagus nerve studies being conducted under acute or semi-chronic conditions. Signals have been recorded acutely from the vagus nerve using extraneural cuff electrodes; however, these electrodes tend to have low SNR due to the insulating perineurium attenuating the neural signals, and restricting recordings to the outer layers of axons. Additionally, vagal recordings have only been carried out under the influence of anesthesia, which is known to alter autonomic activity. Thus, we aimed to record intraneural vagal signals chronically in the rat using CNTY electrodes, both with and without anesthesia. We have previously described implantation of these electrodes by winding the CNTY around a tungsten microneurography needle for anesthetized recording; here, we describe a novel method which simplifies electrode preparation and implantation while increasing successful surgical implants. The stability of these electrodes paired with an improved recording setup also allow for the first ever recordings of vagal activity from awake, behaving rats. Figure 1 shows implantation of the electrodes using this novel suture method, and Fig. 2a shows the chronic awake recording system.

Vagal activity was recorded in anesthetized and awake, behaving rats for up to 11 weeks using custom-ultra-low noise electronics and connector headcap (see Fig. 2a). The recorded activity varied in both awake and anesthetized rats, and included large spontaneous spiking activity (individual spikes as well as bursting). Figure 2b shows an example of baseline activity; the red box represents estimated peak-to-peak noise based on electrode impedance and input amplifier noise^34^. Spiking activity occurred both with and without anesthesia, but was more common in awake animals. Figure 2c,d show examples of short and long spike bursts, while Fig. 2e shows large spiking activity, including individual spikes and short bursts with > 100 µV peak-to-peak voltage. Spikes were detected via thresholding (6 times the baseline RMS), and peak-to-peak spike amplitude was compared to baseline RMS to calculate SNR. Average spike SNR was found to be 16.9 ± 5.4. Figure 2f shows an example of the simultaneously recorded ECG, with Fig. 2g showing the associated heart rate. All of these activity types were observed both with and without anesthesia, though spiking, spike bursting, and large spikes were more common without anesthesia, while quiet baseline was more common with anesthesia. Due to the many organs innervated by the vagus nerve, decoding the function of individual spikes or groups of spikes is a significant challenge. However, average vagal activity can be calculated to directly compare measured vagal tone to HRV.

### Tonic vagal activity is not correlated with HRV

While there is some controversy surrounding the physiological significance of HRV measurements, they are accepted by many as a viable clinical method to estimate parasympathetic (or vagal) and/or sympathetic tone. However, there is very little understanding about how well HRV actually represents overall vagal activity. Using intraneural CNTY electrodes, we measured tonic vagal activity in the left cervical vagus chronically. Vagal ENG and ECG were recorded simultaneously for 10 min while animals were maintained at 2% isoflurane. Electrode noise was estimated for each recording from electrode impedance (Johnson noise) and input amplifier noise. This electrode noise was subtracted from the average RMS for each 10-min recording to obtain a measurement of vagal tone. Across all animals, vagal tone under isoflurane anesthesia was 1.03 ± 0.48 µV RMS (63 recordings from 6 animals). Commonly used HRV metrics were calculated from the ECG recording using ADInstruments’ Labchart HRV module. HRV metrics used were standard deviation of the R–R interval (SDRR), standard deviation of the heart rate (SD Rate), the root mean square of successive differences (RMSSD), high frequency (HF) power, HF power as a percentage of total power (HF%), and low frequency to high frequency power ratio (LF/HF). The high frequency range was defined as 0.5–2 Hz, while the low frequency range was 0.2–0.5 Hz. These 6 metrics were chosen as they are the most common measures in both time and frequency domains. Other candidate metrics (such as nonlinear metrics) were not used due to their high correlation with one of the 6 measures used. Measurements were taken from 1–11 weeks after implantation in 6 rats, and the Pearson correlation and significance between tonic neural RMS and the HRV metrics was calculated for each animal; *p* values and average correlation coefficients for all animals are shown in Table 1. Supplemental Table 1 shows correlation coefficients and correlation *p* values for each animal separately, and Supplemental Fig. 1 shows a scatterplot of the tonic vagal activity with each HRV metric investigated for one animal. None of the HRV metrics were found to have a correlation significantly different from zero across all 6 animals.

**Table 1:** Correlations of anesthetized baseline vagal activity with HRV.

Correlation between anesthetized baseline vagal activity and HRV						
	SDRR	CVRR	RMSSD	HF	HF%	LF/HF
Correlation Coefficients	− 0.13 ± 0.35	− 0.12 ± 0.34	− 0.19 ± 0.37	− 0.044 ± 0.38	− 0.0026 ± 0.50	0.072 ± 0.27
*p* value	0.40	0.42	0.90	0.79	0.98	0.54

Data shown are the averages for six animals (63 total recordings). None of the six HRV metrics have correlations significantly different from zero (Bonferroni corrected significant level of *p* = 0.0083). Supplemental Table 1 shows individual correlations and *p* values for each animal.

Anesthesia is known to significantly change HRV, with isoflurane generally causing a decrease in HRV metrics associated with vagal activity. Previous studies have shown that generalized vagal activity is decreased by anesthesia; at the same time, individual fiber types display varied behavior, with both increases and decreases in firing. Due to the ambiguity of the relationship between vagal activity, HRV, and isoflurane anesthesia, we investigated the correlation between HRV and tonic vagal activity without anesthesia. Non-anesthetized recordings were collected from 4 animals, repeating the measurements described above to determine if tonic vagal activity was correlated with HRV under these conditions. In this case, vagal RMS was averaged over 3–10 min periods, with recordings conducted for 1–4 h. Across all animals, vagal tone without anesthesia was 2.38 ± 2.081 µV RMS (358 recordings from 4 animals) – a significant increase over anesthetized vagal tone (two-sample t-test, *p* = 5.3E−7). Hedge’s *g* was used to measure the effect size of increased vagal tone without isoflurane, yielding a medium effect size with g = 0.70. *P* values and average correlation coefficients between vagal tone and HRV for are shown in Table 2. Supplemental Table 2 shows correlation coefficients and correlation *p* values for each animal separately, and Supplemental Fig. 2 shows a scatterplot of the tonic vagal activity with each HRV metric investigated for one animal. Once again, none of the HRV metrics were found to have a correlation significantly different from zero across all 4 animals, though rat #6 did have significant correlations for several HRV measures (SDRR, CVRR, and LH/HF—positive correlation, HF%—negative correlation). From these measurements, we conclude that tonic vagal activity in both awake and in anesthetized animals has no consistent correlation with any HRV metrics, suggesting that HRV is not a valid estimate of cervical vagal tone, with or without anesthesia.

**Table 2:** Correlations of non-anesthetized baseline vagal activity with HRV.

Correlation between non-anesthetized baseline vagal activity and HRV						
	SDRR	CVRR	RMSSD	HF	HF%	LF/HF
Correlation Coefficients	0.19 ± 0.23	0.26 ± 0.24	0.088 ± 0.10	0.028 ± 0.070	− 0.077 ± 0.17	0.069 ± 0.22
*p* value	0.20	0.12	0.19	0.49	0.44	0.58

Data shown are the averages for four animals (358 total recordings). None of the six HRV metrics have correlations significantly different from zero (Bonferroni corrected significant level of *p* = 0.0083). Supplemental Table 2 shows individual correlations and *p* values for each animal.

### Average vagal activity is increased during inspiration compared to expiration

**Figure 3:**
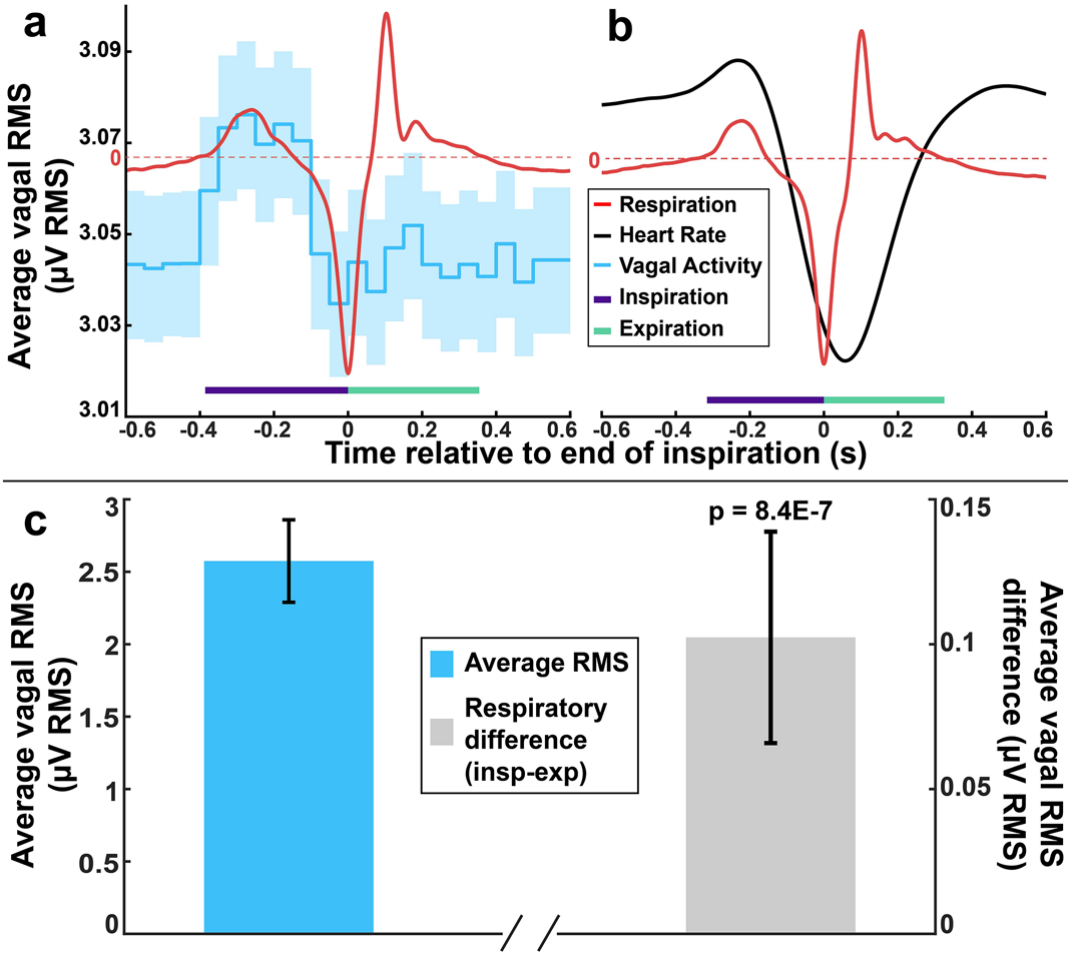
Average vagal activity measured during respiration, under anesthesia. (**a**) Sample dataset obtained from a 10-min recording showing the average accelerometer trace (red, used to measure inspiration and expiration phases), as well as average vagal RMS (blue, with shaded area representing the 95% confidence interval). (**b**) Sample dataset obtained from another 10-min recording showing the average accelerometer trace as well as the average heart rate (black). (**c**) Average values for the total anesthetized RMS (left) and the RMS difference between inspiration and expiration phases (right) using accelerometer to signal respiration (61 recordings total from 4 animals). The respiratory difference is 0.10 ± 0.15 µV RMS, which is significantly different from zero (*p* = 8.4E−7).

The vagal/parasympathetic components of HRV are thought to arise from respiratory sinus arrhythmia, whereby inspiration and expiration cause natural changes in heart rate (heart rate typically increases during inspiration and decreases during expiration). Thus, HRV may not be a measure of tonic vagal activity, but rather phasic activity of the vagus nerve that is modulated with respiration. Coherent averaging is a technique which can be applied to increase SNR of a periodic signal by averaging the recording power based on a specific control trigger. Here, we use a trigger based on respiration to detect changes in vagal activity during different phases of respiration. An accelerometer attached to the torso of the animal was used to measure respiration under anesthesia while simultaneously recording ENG and ECG. Figure 3a shows an example of average vagal RMS (50 ms bin size) alongside the average respiratory trace recorded by the accelerometer for a 10-min recording period (497 breaths). Under isoflurane anesthesia, we observed a reversal of normal RSA. This effect is shown in Fig. 3b, where heart rate decreases during inspiration and increases during expiration. Additionally, we found that average vagal RMS was significantly increased during inspiration compared to expiration (Fig. 3c), with paired t-test of 61 recordings from 4 animals yielding a *p* value of 8.4E−7, with a medium effect size of Cohen’s d = 0.67. The average RMS during expiration was subtracted from the average during inspiration to obtain a quantitative measure of the phasic respiratory signal. This respiratory vagal difference (RVD), is consistent with predicted and measured vagal activity for lung afferents, and may also include the effect of respiration on other vagal fiber types.

### Respiratory vagal difference can be estimated from the ECG

**Figure 4:**
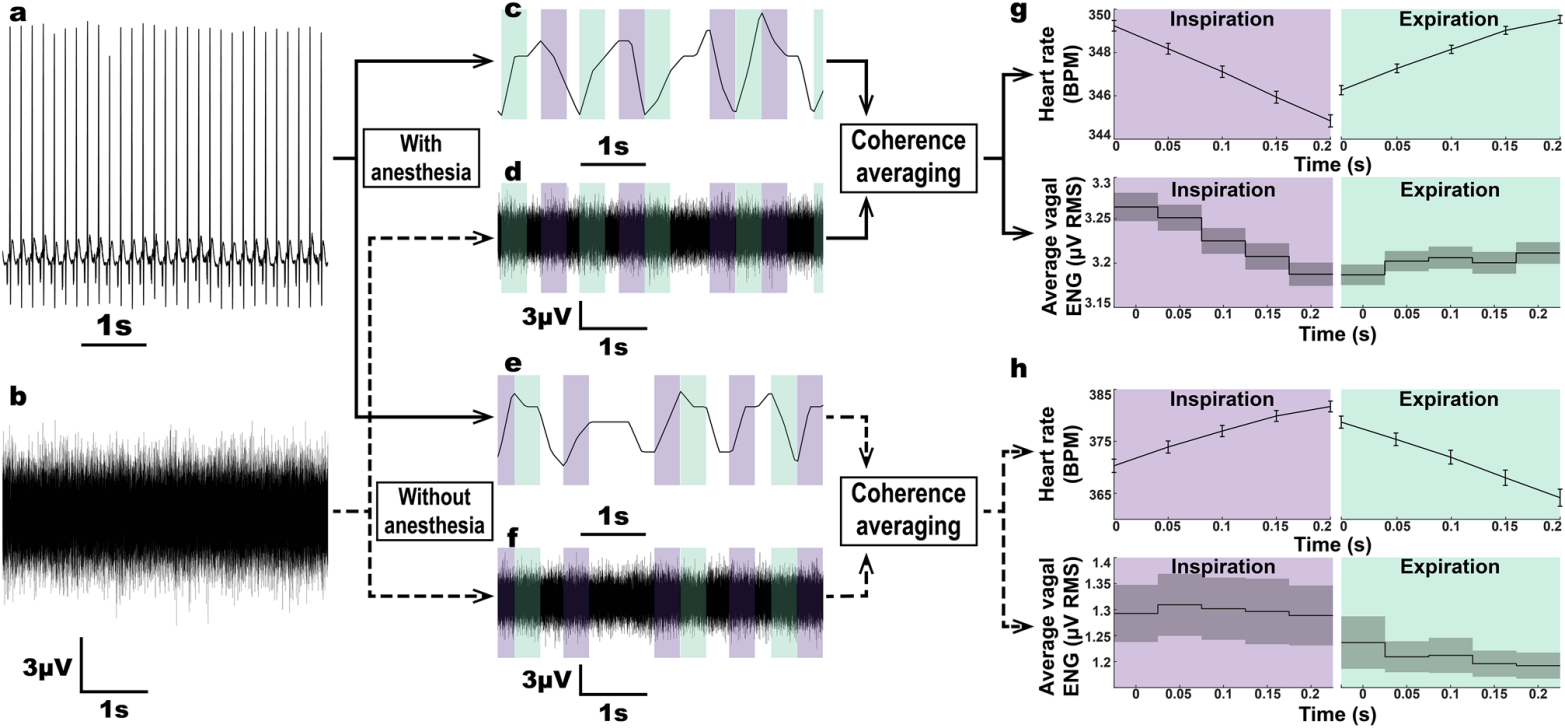
Calculation of respiratory vagal difference (RVD) using heart rate to signal respiration. (**a,b**) Simultaneous recording of ECG (**a**) and vagal ENG (**b**). (**c**) Anesthetized ECG is used to calculate heart rate. Periods of increasing (green—expiration) and decreasing (purple—inspiration) heart rate are identified. (**d**) Filtered vagal ENG is divided into increasing/decreasing heart rate periods. (**e,f**) Steps described in panels (**c**) and (**d**) are repeated for non-anesthetized recordings, but inverted due to RSA reversal under anesthesia. (**g**) Average heart rate and vagal ENG during inspiration and expiration under anesthesia (sample data shown for a single 10-min recording). (**h**) Average heart rate and vagal ENG during inspiration and expiration without anesthesia (sample data shown for a single 10-min recording). RVD is calculated as the difference in vagal RMS between expiration and inspiration over a 10-min period.

**Figure 5:**
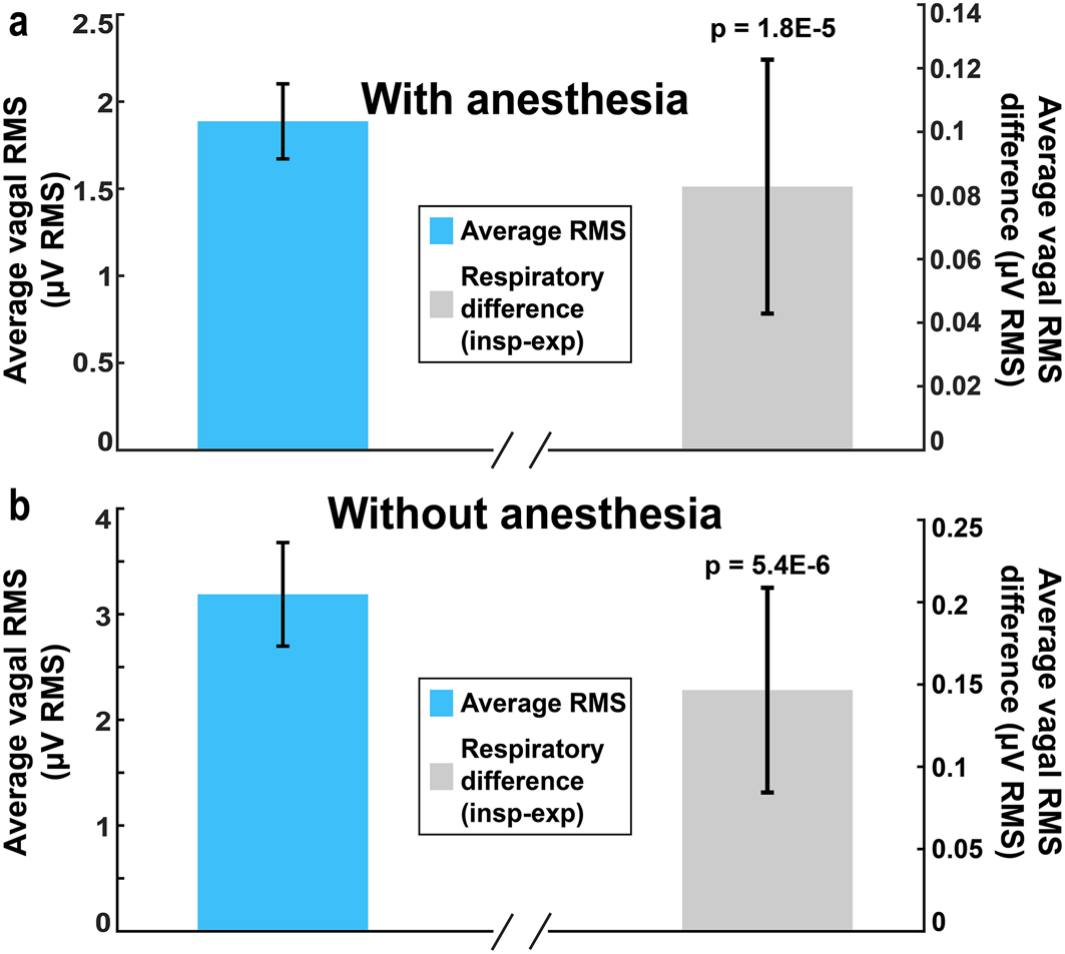
Average tonic RMS and respiratory vagal difference (RVD) using HR to signal respiration. (**a**) With anesthesia: Average RMS and 95% confidence interval in blue, RVD and 95% confidence interval in grey. Average RVD, calculated for 63 total recordings from 6 animals, was 0.0828 ± 0.14 µV RMS, which is significantly different from zero (*p* = 1.8E−5). (**b**) Without anesthesia: Average RMS and 95% confidence interval in blue, RVD and 95% confidence interval in grey. Average RVD, calculated for 63 total recordings from 6 animals, was 0.15 ± 0.60 µV RMS, which is significantly different from zero (*p* = 5.4E−6).

Due to the presence of respiratory sinus arrythmia, it is possible to obtain a measure of respiration from the ECG. We investigated whether RVD could be measured using only the ECG and vagal ENG. Under normal conditions, HR decreases during expiration and increases during inspiration; this is reversed with isoflurane anesthesia. Figure 4 shows how heart rate changes were used to average ENG signal during respiration and estimate RVD both with and without anesthesia. Vagal RMS was calculated in 50 ms bins, and the bins were averaged during periods of heart rate increase (at least 0.25 s long) and heart rate decrease (at least 0.25 s long). Sample data are shown in Fig. 4g,h, while Fig. 5 shows that during inspiration there was an overall increase in vagal activity compared to expiration both with and without anesthesia (paired t-tests, *p* = 1.8E−5, 63 recordings in 6 animals with anesthesia, *p* = 5.4E−6, 358 recordings in 4 animals without anesthesia). Paired sample effect size, Cohen’s *d*, was measured for RVD with and without anesthesia. Under isoflurane anesthesia, RVD has a medium effect size, with d = 0.59, while without anesthesia, there is only a small effect size, with d = 0.24. RVD measured with both the accelerometer and heart rate methods (4 animals, 46 total recordings) showed high correlation between the accelerometer and heart rate methods (R = 0.96, *p* = 2.3E−26; see Supplementary Fig. 3). Thus, RVD can be estimated from the heart rate.

### Respiratory vagal difference does not correlate with HRV

Variability in heart rate is closely related to respiration, with certain HRV metrics specifically measuring respiratory variations. The high frequency components (HF and HF%) specifically measure the power of HRV at respiratory frequencies and are thought to be an accurate representation of vagal tone. Due to this connection between the respiratory cycle and HRV, we hypothesized that the magnitude of RVD (which measures the changes in vagal activity during respiration) could be a better predictor of “vagal tone” (HRV), as compared to tonic activity. RVD, both with and without anesthesia, was compared to average HRV metrics. Tables 3 and 4 show *p* values and average correlations for all animals. Supplemental Tables 3 and 4 show correlations and correlation *p* values for each animal separately, and Supplemental Figs. 4 and 5 show sample scatterplots of the RVD with each HRV metric investigated with and without anesthesia. There are only two animals which show a significant correlation with an individual metric under anesthesia—rat #3, which is negatively correlated with RMSSD, and rat #4, which is positively correlated with HF%. No animals showed a significant correlation with any HRV measures without anesthesia. These data show that there is a clear change in vagal activity during respiration, as indicated by the RVD measurements. However, RVD is not correlated with any HRV metrics, suggesting that HRV is not a valid measure of overall phasic respiratory activity in the vagus.

**Table 3:** Correlations of anesthetized respiratory vagal difference with HRV.

Correlation between anesthetized respiratory vagal activity and HRV						
	SDRR	CVRR	RMSSD	HF	HF%	LF/HF
Correlation Coefficients	0.041 ± 0.49	0.11 ± 0.53	0.042 ± 0.49	− 0.035 ± 0.26	0.011 ± 0.64	− 0.16 ± 0.52
*p* value	0.84	0.62	0.84	0.76	0.70	0.49

Data shown are the averages for six animals (63 total recordings). None of the six HRV metrics have correlations significantly different from zero (Bonferroni corrected significant level of *p* = 0.0083). Supplemental Table 3 shows individual correlations and *p* values for each animal.

**Table 4:** Correlations of non-anesthetized respiratory vagal difference with HRV.

Correlation between non-anesthetized respiratory vagal activity and HRV						
	SDRR	CVRR	RMSSD	HF	HF%	LF/HF
Correlation coefficients	0.10 ± 0.092	0.11 ± 0.10	0.069 ± 0.095	0.0029 ± 0.086	− 0.081 ± 0.15	− 0.084 ± 0.12
*p* value	0.11	0.11	0.24	0.95	0.36	0.26

Data shown are the averages for four animals (358 total recordings). None of the six HRV metrics have correlations significantly different from zero (Bonferroni corrected significant level of *p* = 0.0083). Supplemental Table 4 shows individual correlations and *p* values for each animal.

## Discussion

Bioelectronic medicine and autonomic therapy are rapidly growing fields, and technologies to interface with small autonomic nerves will be crucial to further the research in these disciplines. While previous approaches have had limited success with long-term recording, CNTY electrodes provide a stable, high-SNR interface for chronic peripheral nerve interfacing^33^. With novel techniques for electrode manufacture and preparation described here, CNTYs are now easier to use and more reliable to implant. Anesthesia is also known to alter HRV and individual fiber dynamics^35–43^; yet, vagal recordings have not been obtained under chronic conditions without anesthesia. For the first time, we have shown that vagal tone can be recorded using CNTY electrodes in awake, behaving rats, greatly expanding the scope of possible autonomic studies. Of particular interest to physiologists is the relationship between vagal activity, respiratory sinus arrhythmia and HRV.

Respiratory sinus arrhythmia is a well-known phenomenon present in mammals and other animals that links HRV to respiration^44^. The firing rate of cardiac vagal efferents has been shown to be inversely correlated with heart rate in acute experiments, and can be used to predict changes in heart rate associated with respiratory sinus arrhythmia^45,46^. Vagal activity is also linked to HRV by a correlation between the parasympathetic control of heart rate (measured by the degree of HR increase occurring after vagal blockade) and the peak-to-peak variations in heart rate caused by respiration^25,47^. Initially, studies focused on the cardiac component of the vagus nerve, but since, the measurements of HRV have been used to represent overall vagal or parasympathetic activity. This concept was likely strengthened by studies linking altered HRV and non-cardiac diseases. For example, patients with diabetes have lowered high-frequency HRV^48^. Another study showed decreases in SDRR and normalized high frequency HRV for patients with COPD^20^ and concluded that an increase in vagal activity and lack of sympathetic response to stimulus may contribute to airway obstruction. Multiple studies have reported decreased HRV in patients in critical care, and the recovery of normal HRV is associated with survival and general improvement in children and adults^21^. On the other hand, epileptic patients show an increase in respiratory HRV during interictal periods^12^, demonstrating a potential link to hyperactivity in the brain. As a result, HRV is thought to be representative of overall vagal activity^19^. While it is possible that changes in cardiac vagal activity correspond to changes in the activity of other fibers, our data show that HRV is not representative of total vagal activity, even when averaging across the respiratory cycle.

Vagus nerve stimulation (VNS) is an electronic stimulation therapy used for a variety of diseases, most famously for treatment of drug-resistant (refractory) epilepsy^49^. Despite the assorted successes of VNS, the mechanisms of action are largely unknown. One concept is that stimulating the vagus nerve could increase vagal tone, thus offsetting some negative effects seen in patients. However, conflicting results on the efficacy of VNS in both the right and left vagus to increase HRV have emerged^9,50^. Recently, three major clinical trials were completed with the goal of utilizing VNS to correct autonomic imbalance and improve patient outcomes in heart failure. NECTAR-HF (Boston Scientific), INOVATE-HF (BioControl Medical), and ANTHEM-HF (Cyberonics) all discussed the importance of altered HRV in the study rationale^51–53^, and ANTHEM-HF and NECTAR-HF both measured the effect of VNS on HRV. While both of these studies observed an increase in some of the HRV metrics examined, all three of the clinical trials failed to show clear benefits to patients with VNS treatment^50,54,55^, despite previously promising results in dogs, rats, and humans^56–58^. The ANTHEM-HF trial is the only study which yielded overall positive results, such as improvements in left ventricular ejection fraction. Even so, the authors concede that the placebo effect may have affected their results, and further investigation is necessary. There is a great unmet need to increase our knowledge of the vagus nerve signals, and to answer questions such as: “What is vagal tone?”, “How does vagal activity, or true vagal tone, relate to the clinical measures of HRV?”, and “How can VNS be used to restore autonomic function?”. Direct recording of vagal tone is therefore crucial to improving our ability to study autonomic therapies and evaluating their effectiveness.

The vagus contains a variety of fiber types, targets, and sources, with most being afferent fibers^2^. Thus, tonic vagal activity is likely to contain both afferent and efferent firing which would vary greatly depending on physiological status. We have shown that direct measurement of vagal tone is not correlated with common measures of HRV in rats with and without anesthesia. HRV is used as a predictor for vagal tone in both rodents and humans^59^, therefore this result could be extended to humans and suggests that clinical measures of HRV are not representative of overall vagal activity, and/or that the term “vagal tone” is misleading. Since the effects on vagal blockade and vagotomy on HRV are well-established, it is likely that HRV is associated with the activity of a subset of vagal fibers which modulate their activity in response to physiological changes. Variations in HRV could also occur due to changes in downstream receptors or neurotransmitters, independent of changes in neural activity. Recording signals from the cardiac branches of the vagus would allow for a more thorough investigation of the relationship between cardiac vagal activity and HRV. However, the cardiac branches in the rat are prohibitively small and difficult to access, and the study would likely need to utilize a larger mammalian model. These results do provide a potential explanation for the relative lack of success and consistency of VNS stimulation for heart failure, since stimulation in these studies was a fixed on/off cycle, based on the assumption that changing overall vagal activity was equivalent to increasing HRV. Our results do not explain why VNS has been effective in treating a wide array of other chronic conditions, but they do suggest that these actions are likely taking place through the activation of afferent fibers in the vagus nerve.

Single-unit recordings show that cardiac vagal efferents are highly correlated with respiratory sinus arrhythmia^45–47^. Additionally, pulmonary vagal afferents are correlated with respiration^60^. However, such experiments inherently capture only a small subset of vagal activity, whereas the data presented in this study come from many different locations within the nerve. Acute whole-nerve recordings in the mouse vagus have also shown phasic activity synchronized with respiration^61–63^, a trend which is clearly replicated in these data from the rat vagus. Using coherence averaging to increase the SNR revealed that vagal activity is increased during inspiration relative to expiration. This technique has previously been used with R-peak triggered averaging (based on ECG) to detect changes in vagal activity occurring before acutely-induced seizures^64^. The respiratory vagal difference, RVD, is the average activity of many fibers and indicated a strong correlation between vagal firing and respiration. However, RVD did not significantly correlate with HRV, which further demonstrates that HRV is not an accurate measure of vagal tone. RVD signals originate from many types of vagal fibers, such as cardiac vagal efferents, lung afferents (which may be part of the HRV reflex), and fibers that detect changes in blood pressure. Thus, while phasic activity is the likely driver of HRV, it contains other fibers which are unrelated to cardiac control, explaining why RVD does not correlate directly with HRV.

As the first direct study of vagal tone, this work is somewhat limited in its scope. First, HRV is often measured under non-normal physiological conditions, such as chronic illness. The experiments described here could be repeated using models of chronic disease, such as heart failure, high blood pressure, or diabetes, to determine if vagal tone is altered under those conditions. Physiological interventions, such as exercise, could also be used to illicit known changes in HRV that could be compared to vagal recordings. Pharmacological and electrical stimulation-based therapies should also be investigated, providing new information on how these treatments effect vagal activity acutely and long-term. Although many drugs known to alter vagal tone, such as atropine, primarily work on the receptors rather than the vagal fibers themselves, they may have secondary effects on vagal activity. Furthermore, heart rate is controlled not only by the vagus nerve, but by the sympathetic nervous system as well. There is some evidence that the sympathetic tone can be measured by low frequency HRV, and the sympathovagal balance measured by the LF/HF ratio^65^, though this is a controversial topic^66,67^. The application of chronic recording with CNTY electrodes for both sympathetic and vagal tone could be used to greatly improve our understanding of how the sympathetic and vagal systems interact to control HRV, and how these systems are affected by pathophysiological conditions and stimuli.

Overall, our results indicate that it now possible to study the vagus activity in a chronic animal model and to ask new questions about autonomic physiology. By directly measuring vagal activity, we investigated two measures of “true vagal tone”: (1) average vagal activity, which is the most fitting measure of “tone”, and (2) phasic respiratory activity, which is more closely related to the variations in heart rate. Since neither measure showed significant correlation with HRV metrics, regardless of the presence of isoflurane, we conclude that HRV is not an accurate measure of vagal tone in rats. These results can pave the way for future studies on the exact nature of HRV, and can be used as a basis for investigations of novel autonomic therapy paradigms. Additionally, improvements to the CNTY electrodes and recording setup greatly broadens the research that can be conducted in the peripheral nervous system and can lead to new breakthroughs in the study of the vagus nerve.

## Methods

### Electrode manufacture

CNT yarns were manufactured at Case Western Reserve University as described previously^33^. CNTYs were then mated to stainless steel 35NLT-DFT wire (Fort Wayne Metals) with conductive epoxy resin (H20E, EPO-TEK). Dacron mesh and silicone elastomer (MED-4211/MED-4011, NuSil Silicone Technology) were added to further secure and insulate the CNT-DFT junction. The free end of the CNTY was tied to the end of an 11–0 nylon suture (S&T 5V33) using a fisherman’s knot. The DFT-CNTY-suture assembly was coated with 5 µm parylene C (vapor deposition coating, SMART Microsystems), and then a small section (~ 200 µm long) of insulation was removed using a laser welder (Kelanc Laser). Laser settings were set to 1A current, 0.3 ms pulse width, and 300 µm diameter. Parylene-C removal was confirmed by measuring impedance of the recording site in saline before and after de-insulation (typically ~ 10MΩ before de-insulation, and ~ 10kΩ after). Figure 1a,b show a schematic of the electrode assembly and the de-insulated recording site. Figure 1c,d show close-ups of the CNTY-suture knot and the de-insulated recording site, and Fig. 1e shows an electrode implanted in the rat vagus nerve.

### Surgery

All surgical and experimental procedures were done with the approval and oversight of the Case Western Reserve University Institutional Animal Care and Use Committee (protocol number 2016–0328) to ensure compliance with all federal, state and local animal welfare laws and regulations. Electrodes were implanted in male Sprague–Dawley rats between 7–12 weeks of age.

The left cervical vagus nerve was exposed through a midline incision along the neck. Muscles on the left side of the neck were separated to expose the vagus nerve and carotid artery, which are normally mated together. The vagus nerve was separated from the artery and held in slight tension on a glass hook. Two CNTY electrodes were implanted in the nerve by sewing the suture-CNTY electrode through ~ 1 to 2 mm of the nerve, as shown in Fig. 1e. Electrodes were implanted approximately 2 mm apart. The knot was pulled through the nerve, and then pulled back such that the knot rested against the epineurium and the recording site remained inside the nerve. After implantation, the nerve and surrounding tissue were covered with ~ 1 mL of fibrin glue (Tisseel, Baxter International Inc.) to secure the electrodes in place. The DFT ends of the electrode were tunneled to the top of the skull, and then soldered to a connector (Omnetics Connector Corporation MCP-5-SS) which was fixed to the top of the skull using dental cement. The amplifier ground was connected to a screw placed in the skull. Electrodes were implanted for chronic recording in 8 animals, with implant duration varying from two to eleven weeks. Early termination of the experiment sometimes occurred as a result of skin erosion exposing tunneled leads, or from damage to the headcap connector causing pain or discomfort to the animal.

Implantation of the ECG telemeters was performed as described by Kaha Sciences^68^. Telemeters were implanted in the abdomen and fixed to the abdominal muscle, and ECG leads were tunneled to the chest, with one lead placed on the xyphoid process, and the other placed near the bottom of the sternohyoid muscle. Vagus nerve CNTY implants and ECG telemeter implants were performed during a single surgery to minimize impact on the animal.

### Recording

Recordings were carried out in awake, behaving animals, and in animals anesthetized with isoflurane gas. Four animals underwent recordings under anesthesia only, two animals were recorded without anesthesia only, and two animals were recorded both with and without anesthesia (total of 6 animals with anesthesia and 4 without). For anesthetized recording, animals were anesthetized with 4% isoflurane and maintained at 2% isoflurane with 100% oxygen. A quarter-sized mini-board (shown inset in Fig. 2a) was connected to the headcap. This board amplifies and digitizes the signal (via Intan RHD2216 recording chip) before sending the signal to be displayed and saved on a laptop computer. 8-channel hardware averaging was utilized to further increase SNR^34^. Neural recordings were sampled at 20 kHz with a 5 kHz low pass filter. ECG was simultaneously recorded with a 1 kHz sampling rate using a TR50B Biopotential telemeter (Kaha Sciences) via ADInstruments Powerlab and LabChart software. In some cases, an accelerometer (Adafruit ADXL335) was fixed to the animals’ torso to detect movement that occurred during respiration. For awake recordings, the amplifier board was secured to the headcap connector using a custom-made 3D printed pin-locked mechanism (Supplemental Fig. 6a), and attached to a PlasticsOne commutator which allowed the rat to move freely around the cage, as shown in Fig. 2a. This recording setup is also robust against motion of the animal and the board, as shown in Supplemental Fig. 6b. During recordings, the telemeter charging field was turned off as much as possible due to significant interference with the ENG signal. Periods where the telemeter charging field was active, or where ECG signal was poor (due to packet loss from the telemetry, or EMG contamination), were excluded from the analysis.

### Signal processing

ENG, ECG, and accelerometer data were imported into MATLAB, where they were further processed. ENG was band pass filtered from 800–5000 Hz to minimize interference from EMG, ECG, or other possible sources; ECG was low-pass filtered at 300 Hz. Electrode noise was estimated for each recording from electrode impedance (Johnson noise) and input amplifier noise, as described previously^34^, and this noise estimate was subtracted from average recorded RMS to estimate the vagal tone RMS. ECG data was low-pass filtered with a cutoff of 300 Hz. Instantaneous heart rate was calculated by taking the inverse of successive R–R intervals, and was interpolated to generate a signal with a fixed sampling rate of 1 kHz. HRV metrics were calculated using the Labchart HRV module. Beat detection typically including beats with 150–250 ms RR interval and 0–1.8 complexity (though these values were adjusted slightly between animals and recording days as needed). Ectopic beats were excluded from analysis, and recordings with more than 10% ectopic beats or artifacts were excluded, with artifacts and high noise (present only in non-anesthetized recordings) being the most common reasons for exclusion. Data were analyzed in continuous sections between 3 and 10 min long, or approximately 1000–3000 RR intervals.

### Statistical methods

Results in text are reported as mean ± standard deviation, while error bars and shaded areas represent the 95% confidence interval (mean ± 1.96*SEM). T-tests were used to measure significance of correlations, with a Bonferroni corrected significance level of 0.0083; paired t-tests were used to measure significance of respiratory vagal difference (comparing inspiration to expiration), and a two-sample t-test was used to compare the average vagal tone for anesthetized to non-anesthetized recording. All reported *p* values are two-tailed.

## Supplementary Information

Supplementary Information.

## Data Availability

The data that support the findings of this study are available from the corresponding author upon reasonable request. Some custom analysis code was created to calculate and measure RVD and is available from the corresponding author upon reasonable request.
