# Author Correction: Direct measurement of vagal tone in rats does not show correlation to HRV

**DOI:** 10.1038/s41598-021-88882-5

**Published:** 2021-05-07

**Authors:** Joseph T. Marmerstein, Grant A. McCallum, Dominique M. Durand

**Affiliations:** Department of Biomedical Engineering, Case Western Reserve University, Cleveland, OH USA

Correction to: *Scientific Reports*, 10.1038/s41598-020-79808-8, published online 13 January 2021.

The original version of this Article contained errors.

In the Results section, subheading “Average vagal activity is increased during inspiration compared to expiration”,

“The vagal/parasympathetic components of HRV are thought to arise from respiratory sinus arrhythmia, whereby inspiration and expiration cause natural changes in heart rate (heart rate typically increases during expiration and decreases during expiration).”

now reads:

“The vagal/parasympathetic components of HRV are thought to arise from respiratory sinus arrhythmia, whereby inspiration and expiration cause natural changes in heart rate (heart rate typically increases during inspiration and decreases during expiration).”

Under the subheading “Respiratory vagal difference can be estimated from the ECG”,

“Under normal conditions, HR increases during expiration and increases during inspiration; this is reversed with isoflurane anesthesia.”

now reads:

“Under normal conditions, HR decreases during expiration and increases during inspiration; this is reversed with isoflurane anesthesia.”

The original Article has been corrected.

