# Supplementary material for: Direct measurement of vagal tone in rats does not show correlation to HRV
Source: Sci Rep. 2021 Jan 13;11:1210. doi: 10.1038/s41598-020-79808-8 (PMC7807082; doi:10.1038/s41598-020-79808-8)
Supplement: Supplementary file 1 — Supplementary Information. [file 41598_2020_79808_MOESM1_ESM.pdf]

Direct measurement of vagal tone in rats does not show correlation to HRV

Joseph T Marmerstein, Grant A McCallum, Dominique M Durand*
jtm124@case.edu gam19@case.edu dxd6@case.edu*

Affiliations: Case Western Reserve University Biomedical Engineering

Key words: vagus nerve, vagal tone, heart rate variability, intraneural, intrafascicular, recording, carbon nanotube

Supplemental Figures and Tables

Correlation between anesthetized tonic vagal activity and HRV for each animal													
**Animal Number**	**N (recordings per animal)**	**SDRR**		**CVRR**		**RMSSD**		**HF**		**HF%**		**LF/HF**	
		**p**	**R**	**p**	**R**	**p**	**R**	**p**	**R**	**p**	**R**	**p**	**R**
**1**	**7**	0.23	-0.52	0.20	-0.55	0.50	-0.31	0.68	-0.20	0.77	0.14	0.58	0.26
**2**	**15**	0.69	0.11	0.68	0.12	0.43	0.22	0.75	0.090	0.63	-0.13	0.42	-0.23
**3**	**17**	0.45	0.20	0.41	0.21	0.14	-0.38	0.38	-0.23	0.27	0.28	0.25	-0.30
**4**	**9**	0.67	0.16	0.99	0.0029	0.13	0.55	0.045	0.68	0.76	-0.12	0.54	0.24
**5**	**8**	0.12	-0.60	0.15	-0.56	0.50	-0.28	0.44	-0.32	0.46	-0.31	0.41	0.34
**6**	**7**	0.76	-0.14	0.94	0.035	0.85	0.087	0.53	-0.29	0.78	0.13	0.81	0.11

Supplemental Table 1: Individual correlations and p-values of anesthetized baseline vagal activity with heart rate variability for six animals. None of the conditions have correlations significantly different from zero (Bonferroni corrected significant level of p = 0.0083).

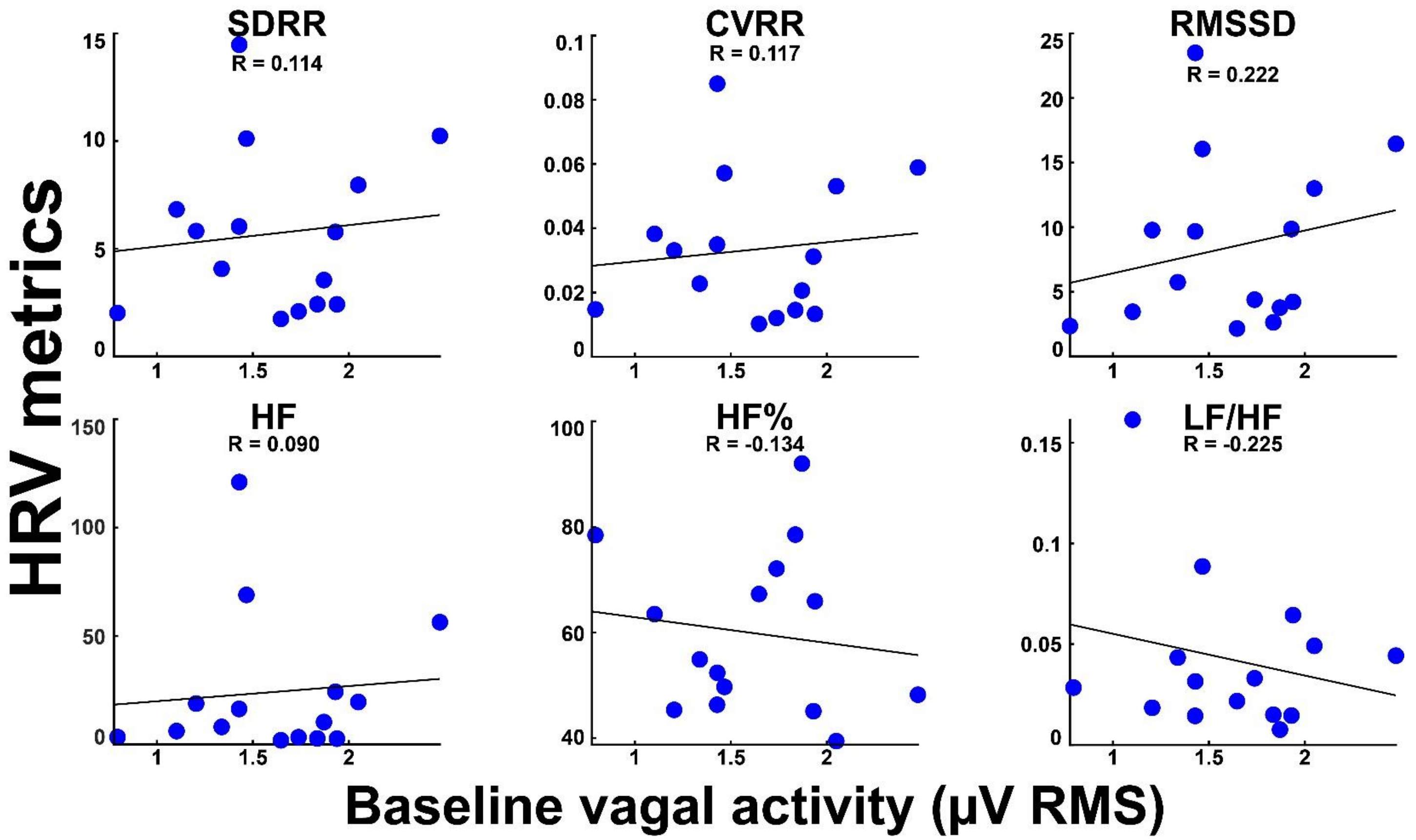

Supplemental Figure 1: Correlation of anesthetized baseline vagal activity with heart rate variability for animal #2 (10 minute recordings taken over 15 different days). None of the HRV measures have a significant correlation with tonic vagal activity.

Correlation between non-anesthetized tonic vagal activity and HRV for each animal													
**Animal Number**	**N (recordings per animal)**	**SDRR**		**CVRR**		**RMSSD**		**HF**		**HF%**		**LF/HF**	
		**p**	**R**	**p**	**R**	**p**	**R**	**p**	**R**	**p**	**R**	**p**	**R**
**4**	**132**	0.33	0.085	0.052	0.17	0.83	0.019	0.96	-0.0045	0.42	-0.070	0.43	0.069
**6**	**99**	**9.4E-8**	**0.51**	**2.9E-10**	**0.58**	0.35	0.096	0.35	0.095	**0.0017**	**-0.31**	**7.6E-6**	**0.34**
**7**	**75**	0.052	0.23	0.015	0.28	0.044	0.23	0.51	0.077	0.82	-0.027	0.080	-0.20
**8**	**52**	0.78	-0.040	0.98	0.0028	0.97	0.0054	0.70	-0.056	0.48	0.10	0.63	0.069

Supplemental Table 2: Individual correlations and p-values of non-anesthetized baseline vagal activity with heart rate variability for four animals. Rat 6 had significant correlations between baseline activity and several HRV metrics (highlighted in green).

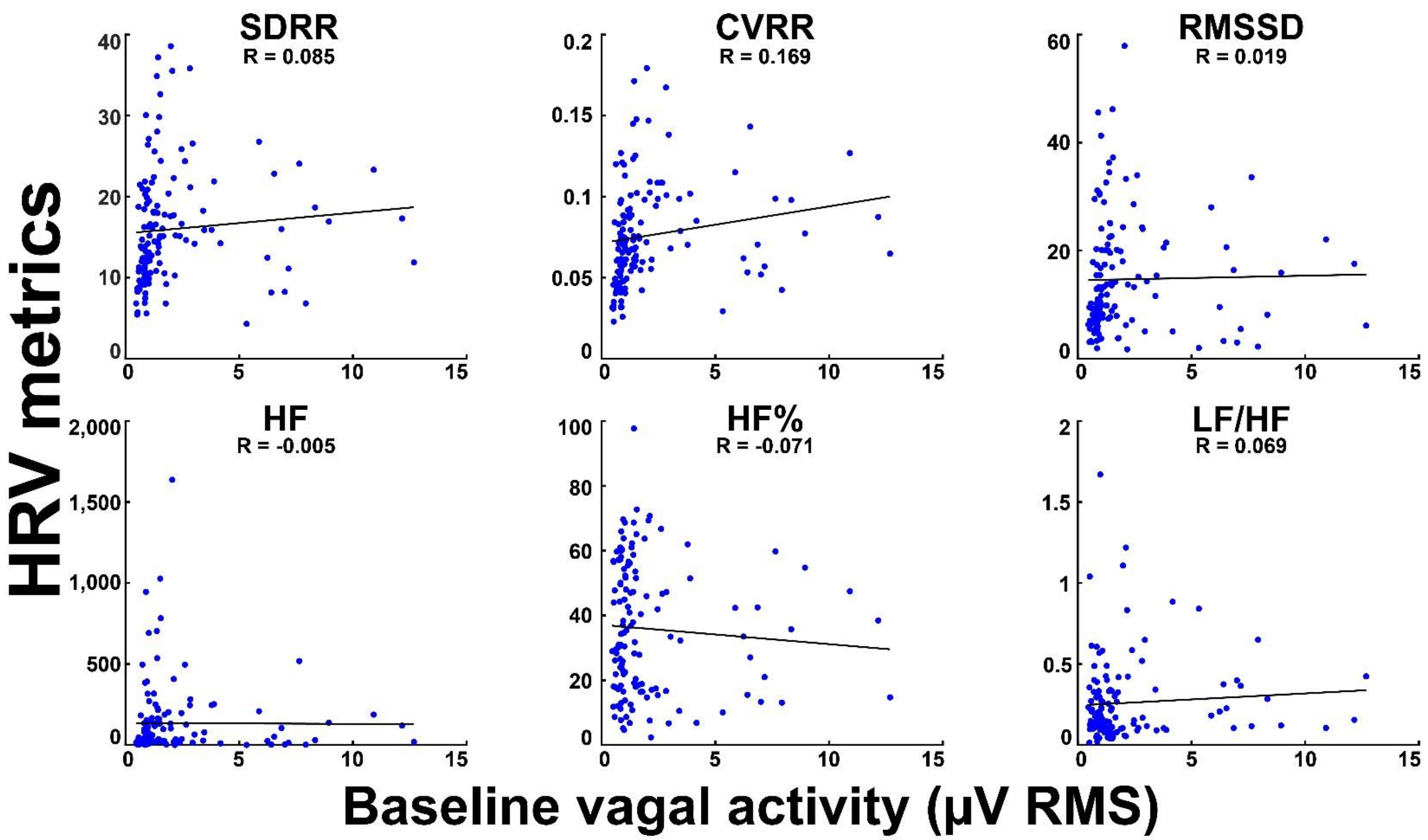

Supplemental Figure 2: Correlation of non-anesthetized baseline vagal activity with heart rate variability for animal #4 (132 5-10 minute recordings taken over 10 different days). None of the HRV measures have a significant correlation with tonic vagal activity.

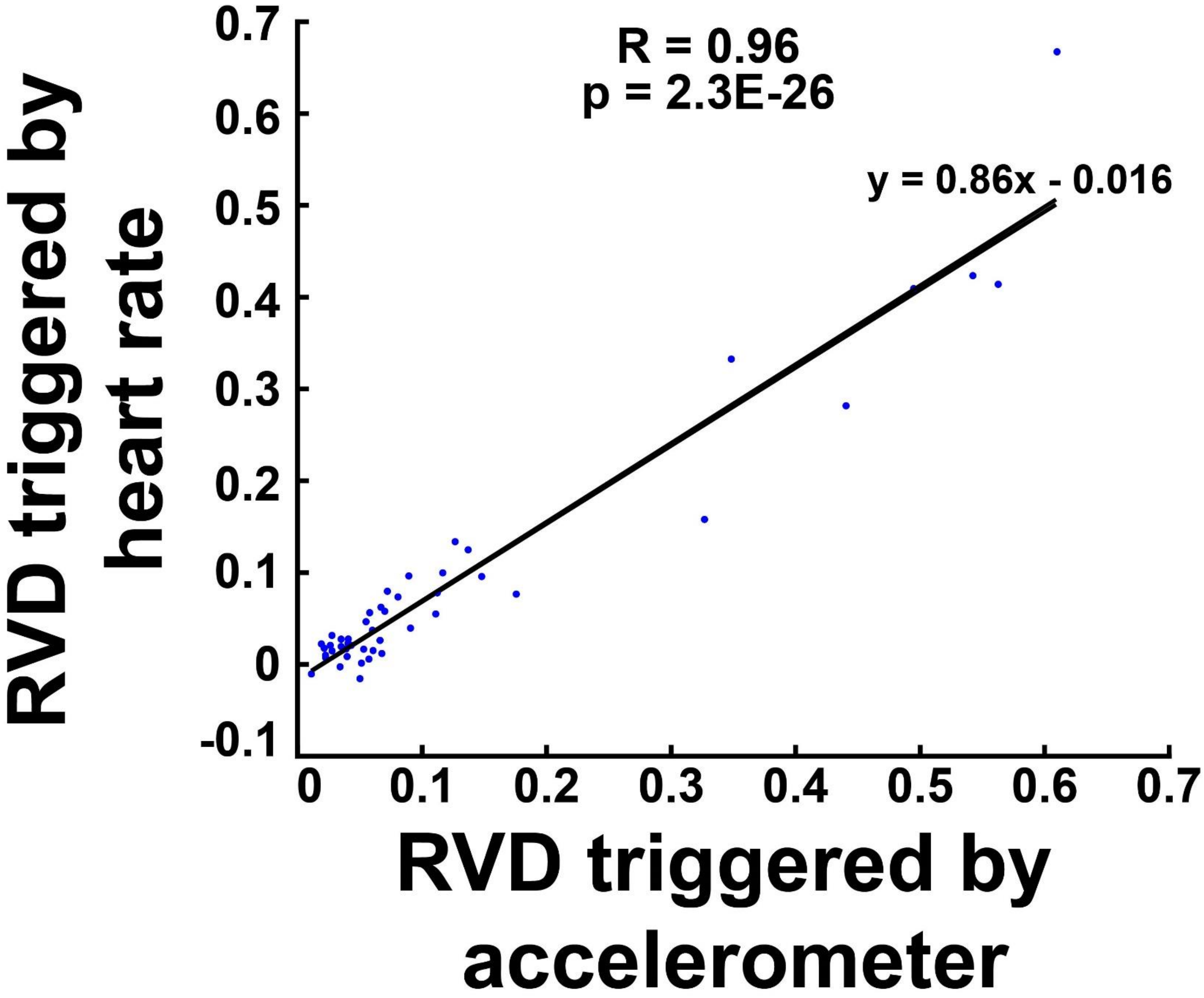

Supplemental Figure 3: Correlation between two different RVD measurements. On the x-axis, respiration averaging is triggered by an accelerometer which measures the movement of the torso during breathing. On the y-axis, respiration averaging is triggered by changes in the heart rate, which varies during respiration. The two methods have a very high correlation (R = 0.96, p = 2.3E-26).

Correlation between anesthetized tonic vagal activity and HRV for each animal													
**Animal Number**	**N (recordings per animal)**	**SDRR**		**CVRR**		**RMSSD**		**HF**		**HF%**		**LF/HF**	
		**p**	**R**	**p**	**R**	**p**	**R**	**p**	**R**	**p**	**R**	**p**	**R**
**1**	**7**	0.064	-0.73	0.088	-0.69	0.88	-0.074	0.64	0.21	0.21	0.55	0.31	-0.45
**2**	**15**	0.67	-0.12	0.61	-0.14	0.82	-0.063	0.74	-0.093	0.95	0.020	0.16	-0.38
**3**	**17**	0.82	0.060	0.76	0.082	**2.6E-4**	**-0.77**	0.063	-0.46	0.025	0.54	0.034	-0.52
**4**	**9**	0.54	0.24	0.13	0.54	0.53	0.24	0.62	0.19	**0.0059**	**0.83**	0.083	-0.61
**5**	**8**	0.93	0.037	0.89	0.059	0.65	0.19	0.76	0.13	0.087	-0.64	0.20	0.51
**6**	**7**	0.046	0.76	0.019	0.84	0.064	0.73	0.68	-0.19	0.12	-0.65	0.24	0.51

Supplemental Table 3: Individual correlations and p-values of anesthetized respiratory vagal difference with heart rate variability for six animals. Rats 3 and 4 each had a significant correlation with one HRV metric (highlighted in green).

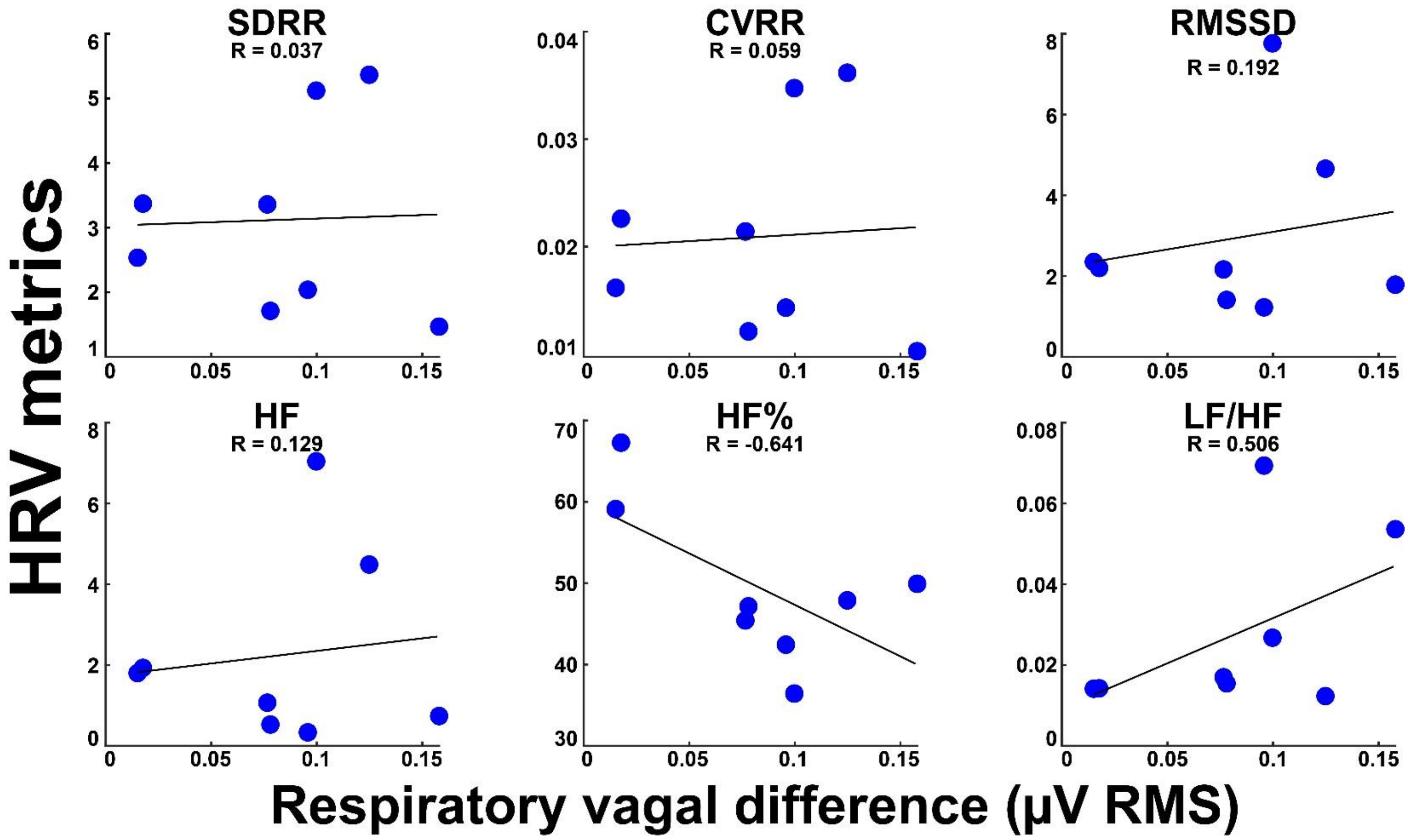

Supplemental Figure 4: Correlation of anesthetized respiratory vagal difference with heart rate variability for animal #5 (10 minute recordings taken over 8 different days). None of the HRV measures have a significant correlation with RVD.

Correlation between non-anesthetized tonic vagal activity and HRV for each animal													
**Animal Number**	**N (recordings per animal)**	**SDRR**		**CVRR**		**RMSSD**		**HF**		**HF%**		**LF/HF**	
		**p**	**R**	**p**	**R**	**p**	**R**	**p**	**R**	**p**	**R**	**p**	**R**
**4**	**132**	0.31	0.090	0.55	0.052	0.15	0.12	0.66	0.039	0.13	0.13	0.029	-0.19
**6**	**99**	0.021	0.23	0.010	0.26	0.11	0.16	0.39	0.097	0.28	-0.11	0.42	0.081
**7**	**75**	0.88	0.018	0.69	0.046	0.66	-0.051	0.38	-0.10	0.064	-0.21	0.19	-0.15
**8**	**52**	0.63	0.068	0.49	0.098	0.77	0.041	0.88	-0.021	0.34	-0.14	0.61	-0.073

Supplemental Table 4: Individual correlations and p-values of non-anesthetized baseline vagal activity with heart rate variability for four animals. None of the conditions have correlations significantly different from zero.

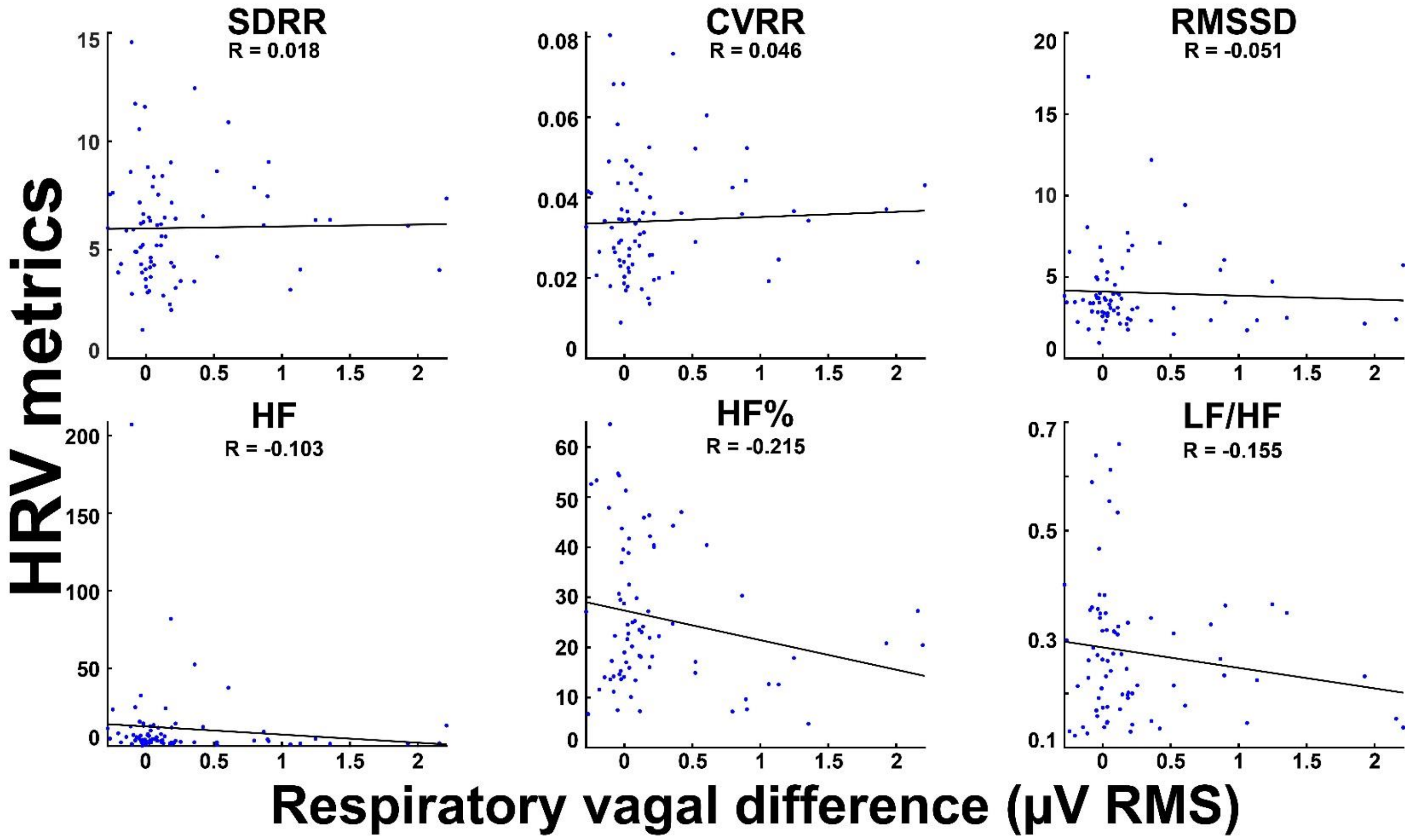

Supplemental Figure 5: Correlation of non-anesthetized respiratory vagal difference with heart rate variability for animal #7 (75 5-10 minute recordings taken over 4 different days). None of the HRV measures have a significant correlation with RVD.

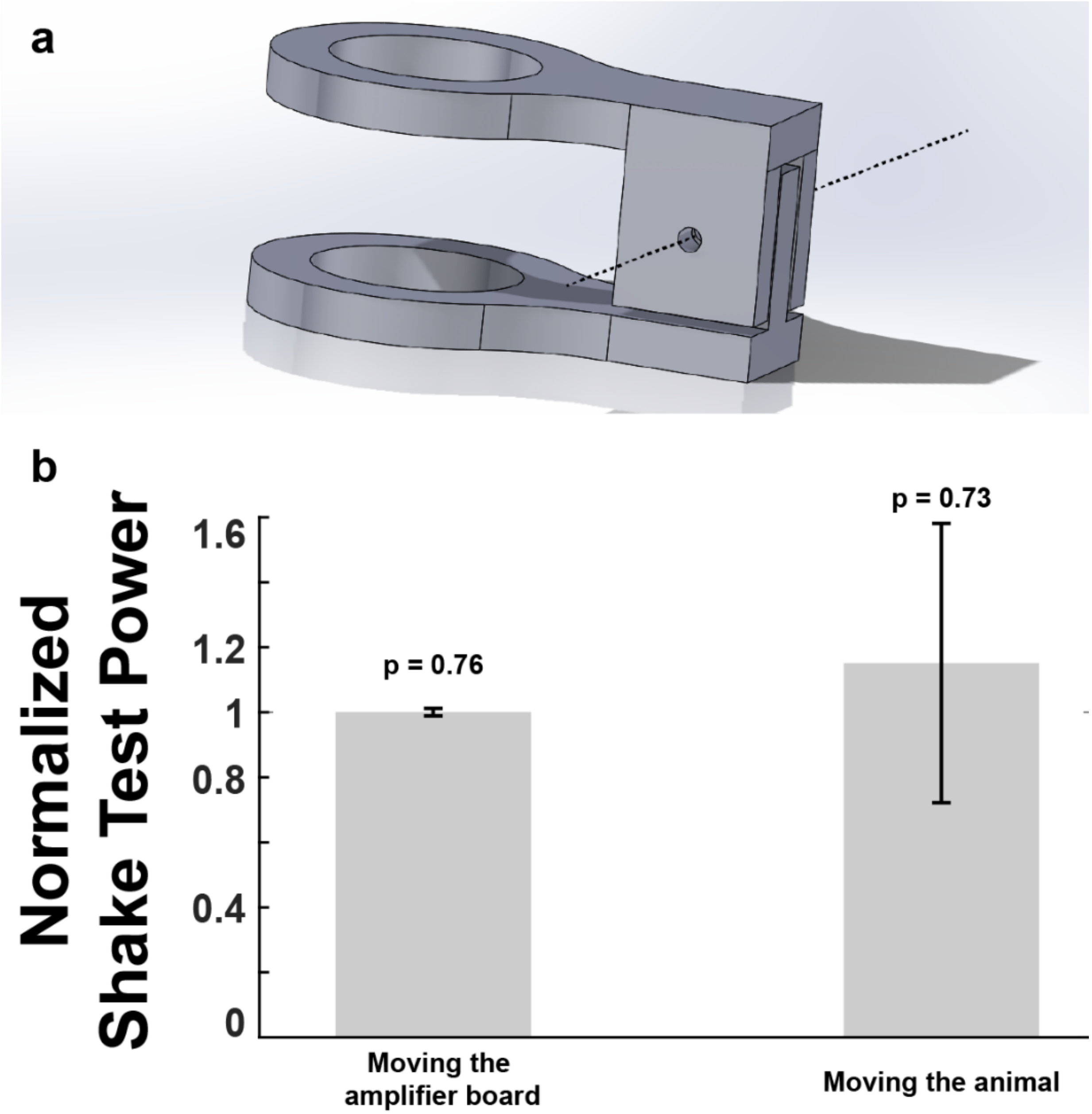

Supplemental Figure 6: a. Schematic of the 3D printed locking mechanism used to secure the amplifier board for awake recordings. Each of the pieces is secured to the two connectors (one on the board, one on the animal). When recording, a safety pin is inserted into the mechanism to prevent the animal from unplugging the board. **b.** Shake tests were conducted to determine if the recordings were robust to movement artifacts. Two types of shake tests were conducted, with average RMS during the shaking (~10-30s) compared to the average RMS of the baseline recorded on the same day. The first shake test involved grabbing the amplifier board and moving it around, while the other involved holding and moving the whole animal. Bar graphs show the shake test RMS (3 experiments in 2 animals) normalized to the baseline for that day, error bars show 95% confidence interval. Neither shake test produced statistically significant changes in average RMS (two-tailed, paired t-test).
